# Supplementary material for: Comparison of Automated and Traditional Western Blotting Methods
Source: Methods Protoc. 2023 Apr 20;6(2):43. doi: 10.3390/mps6020043 (PMC10142486; doi:10.3390/mps6020043)
Supplement: Supplementary file 1 [file mps-06-00043-s001.zip › mps-2317422-supplementary.pdf]

SUPPLEMENTARY
Figure S1 (a)

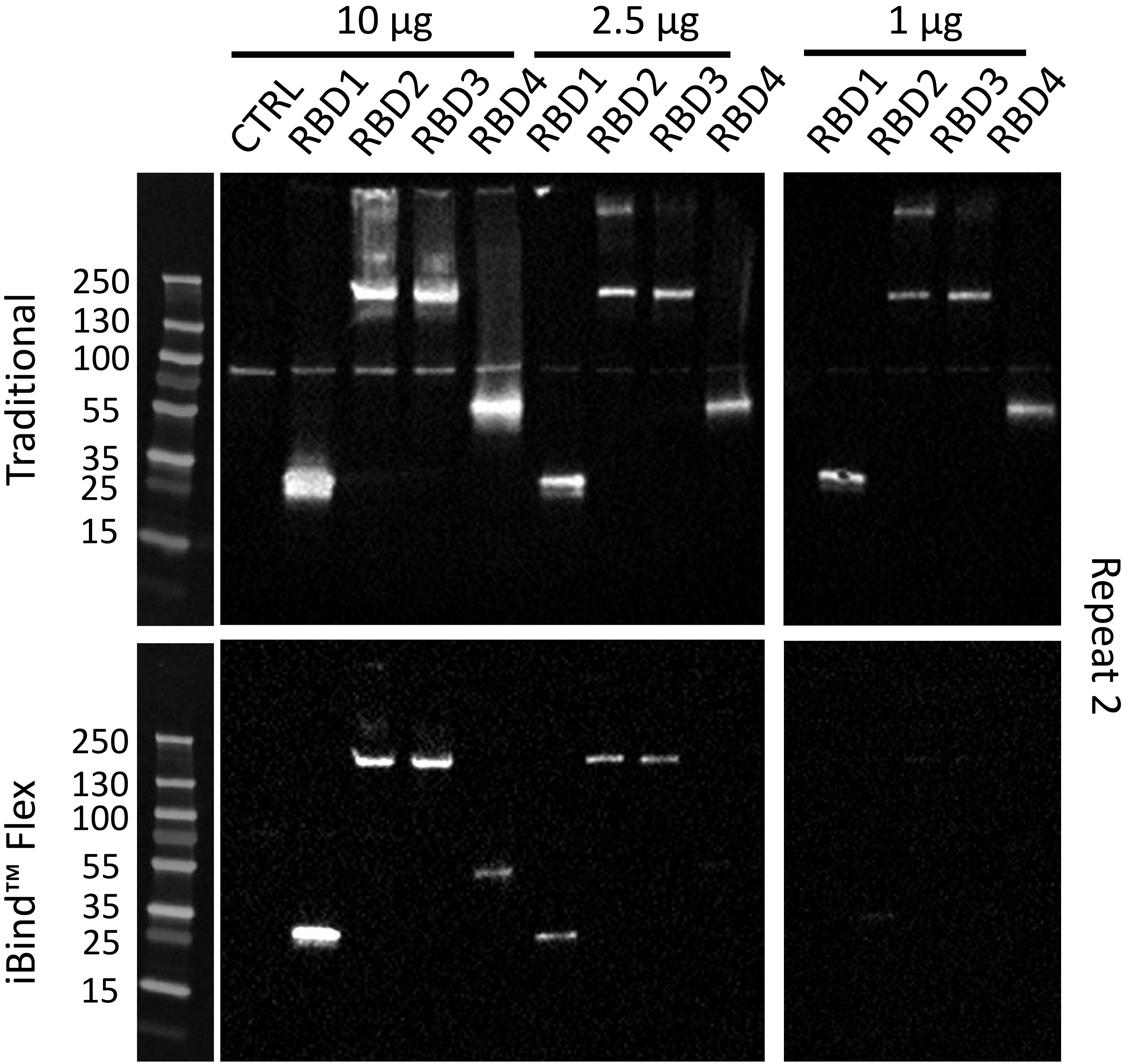

SUPPLEMENTARY
Figure S1 (b)

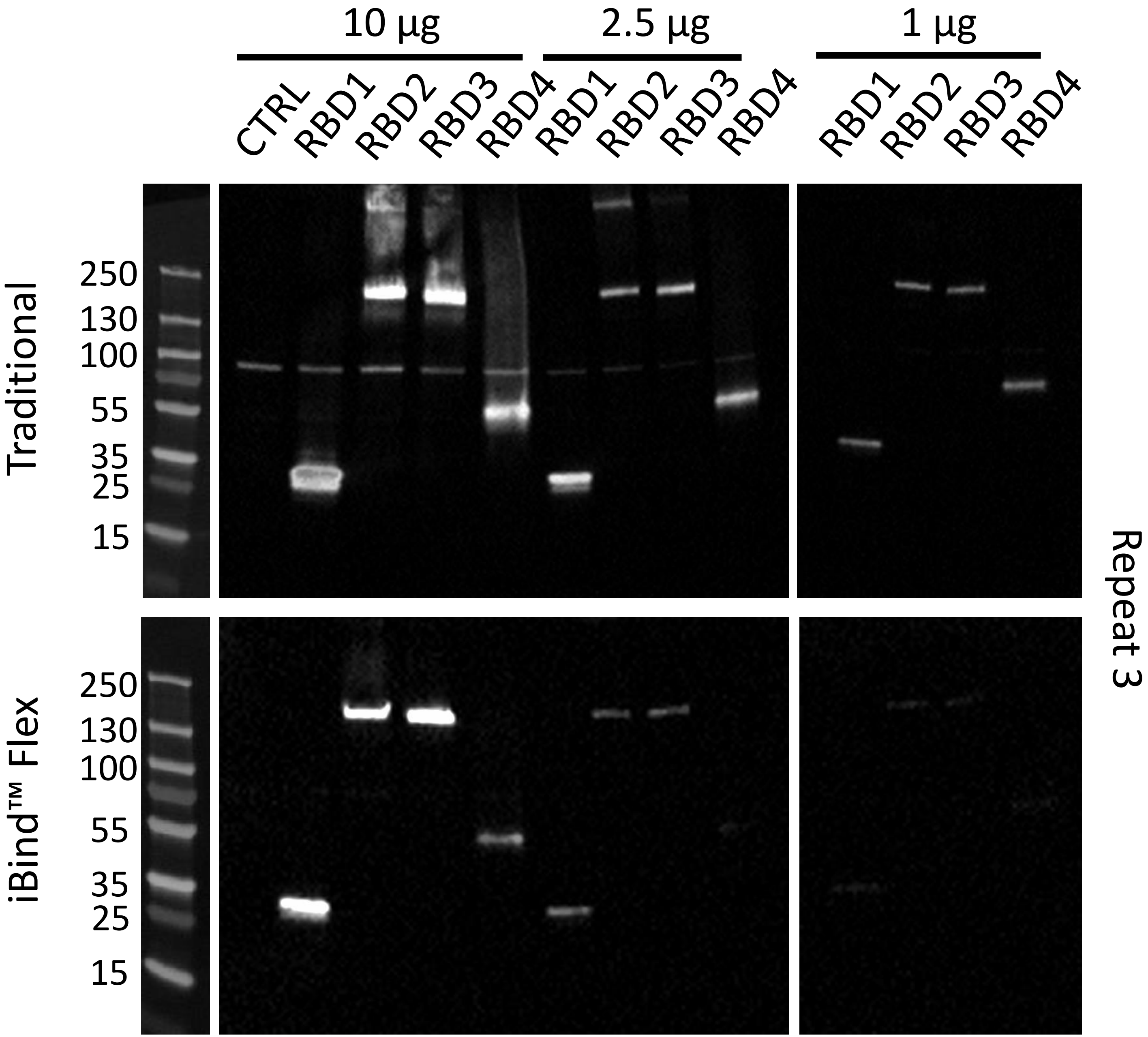

Supplementary Figure S1. Lysates containing RBD proteins analyzed by traditional WB and iBind™ Flex. Image of membranes immunoblotted with anti-SARS-CoV-2 RBD (1:2500) using traditional WB (top) and iBind™ Flex for immunoblotting (bottom) for second (a) and third (b) repeat of experiments. Lysates (10, 2.5 and 1 μg/lane) were from 293T cells transfected with plasmids expressing different-sized transgenes containing RBD (RBD1-4) or control transgene (CTRL). MW: PageRuler™ Plus Prestained Protein Ladder (Thermo Scientific).

SUPPLEMENTARY
Figure S2

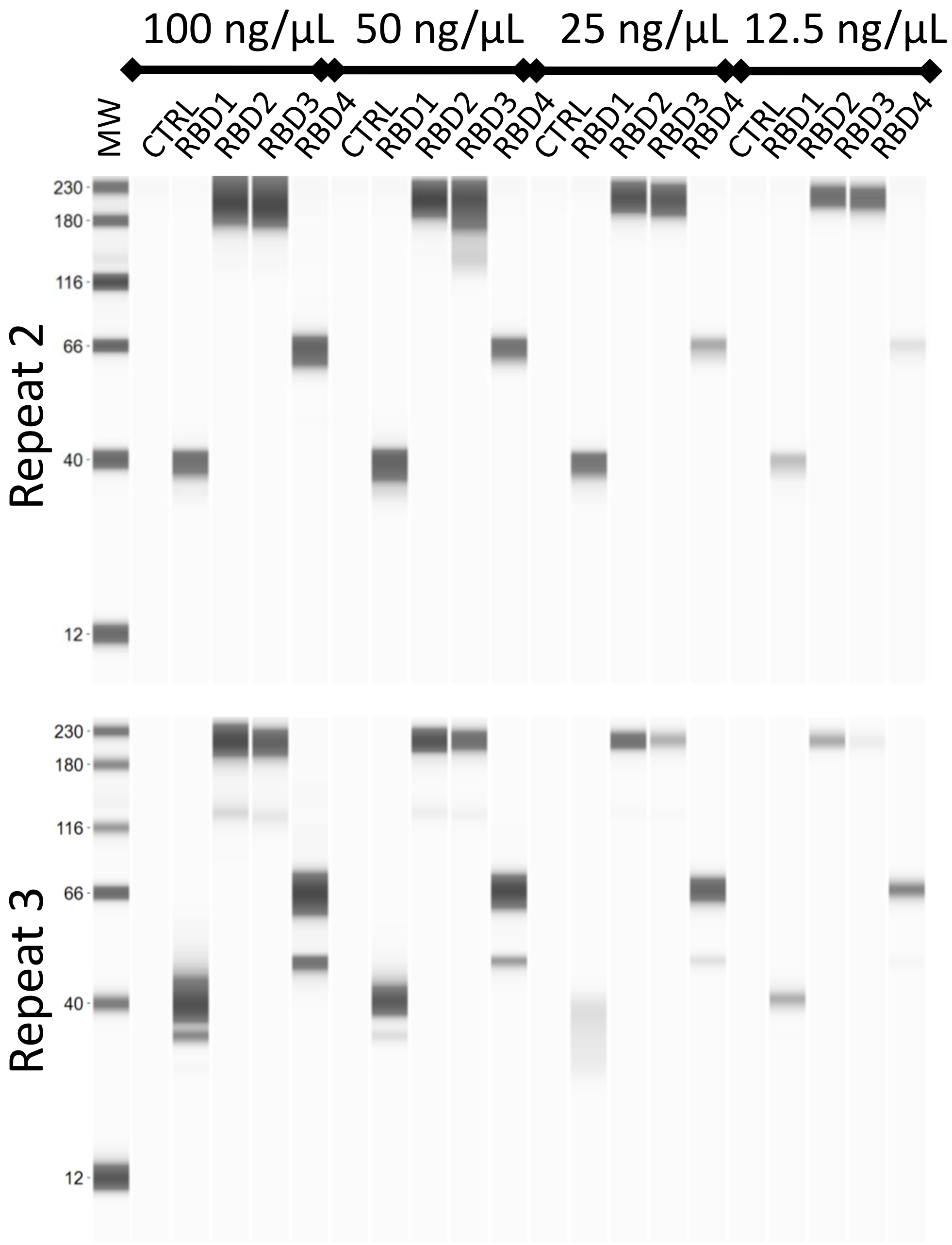

Supplementary Figure S2. Assessment of transgene expression by JESS Simple Western™. Different concentrations (100, 50, 25, 12.5 ng/μL) of lysates from 293T cells transfected with vectors expressing RBD containing transgenes of different sizes (RBD1-4) or control transgene (CTRL) were processed in the fully automated JESS Simple Western™ device, probed with the SARS-CoV-2 RBD (1:50). Portrayal of a traditional blot-like image with a lane-view of the samples for two additional, independent experiments.
